# Supplementary material for: Patients with basal ganglia damage show preserved learning in an economic game
Source: Nat Commun. 2019 Feb 18;10:802. doi: 10.1038/s41467-019-08766-1 (PMC6379550; doi:10.1038/s41467-019-08766-1)
Supplement: Supplementary file 1 — Supplementary Information [file 41467_2019_8766_MOESM1_ESM.pdf]

Supplementary Information

Patients with basal ganglia damage show preserved learning in an economic game
Zhu et al.

Supplementary Figures 1-10
Supplementary Tables 1-6

A. Strategic

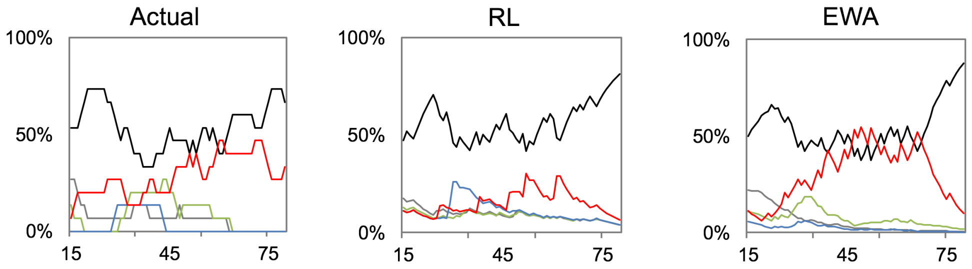

B. Non-Strategic

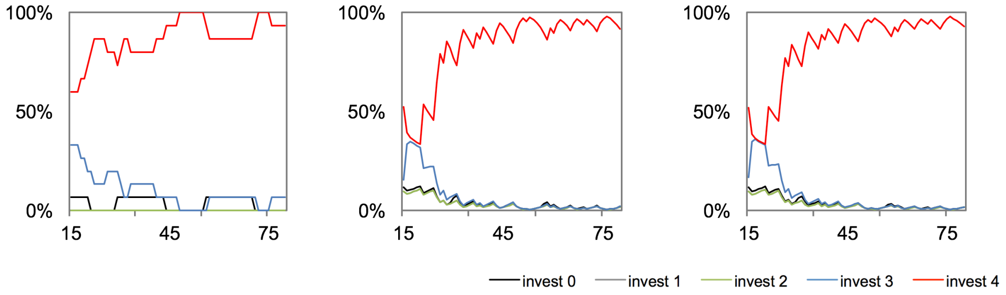

Supplementary Figure 1. Illustration of behavior and model predictions using data from representative healthy participants in the Weak role (i.e., can invest from 0 to 4). Actual time series of choice was plotted by using a 15-round bin average. Choice probabilities were generated using the best-fitting parameters based on the RL and hybrid EWA learning, respectively. **(A)** Strategic condition: The engagement in the belief learning under the strategic condition, which is not captured by RL, is most saliently reflected in a decreased probability of investing 0 and increased probability of 4 in rounds 40-60. This corresponding to periods when Strong players invested 1-2 units with increased likelihood. EWA model fit demonstrates a significant improvement over RL, which fails to account for the strategy shift during this period (BIC difference between EWA and RL = 9.69; EWA pseudo-R^2 = 0.33). **(B)** Non-strategic condition: Both RL and EWA capture the overall pattern of the observed choice dynamics, yielding almost identical sequences of predictions. Formal model comparison suggests that, whereas EWA provided a reasonable fit for the data (EWA pseudo-R^2 = 0.70), it fits worse than RL after penalizing for the additional parameters (BIC difference between EWA and RL = -4.93), consistent with previous findings suggesting learning was primarily driven by reinforcement learning when the information of incentive structure of the opponent was missing.

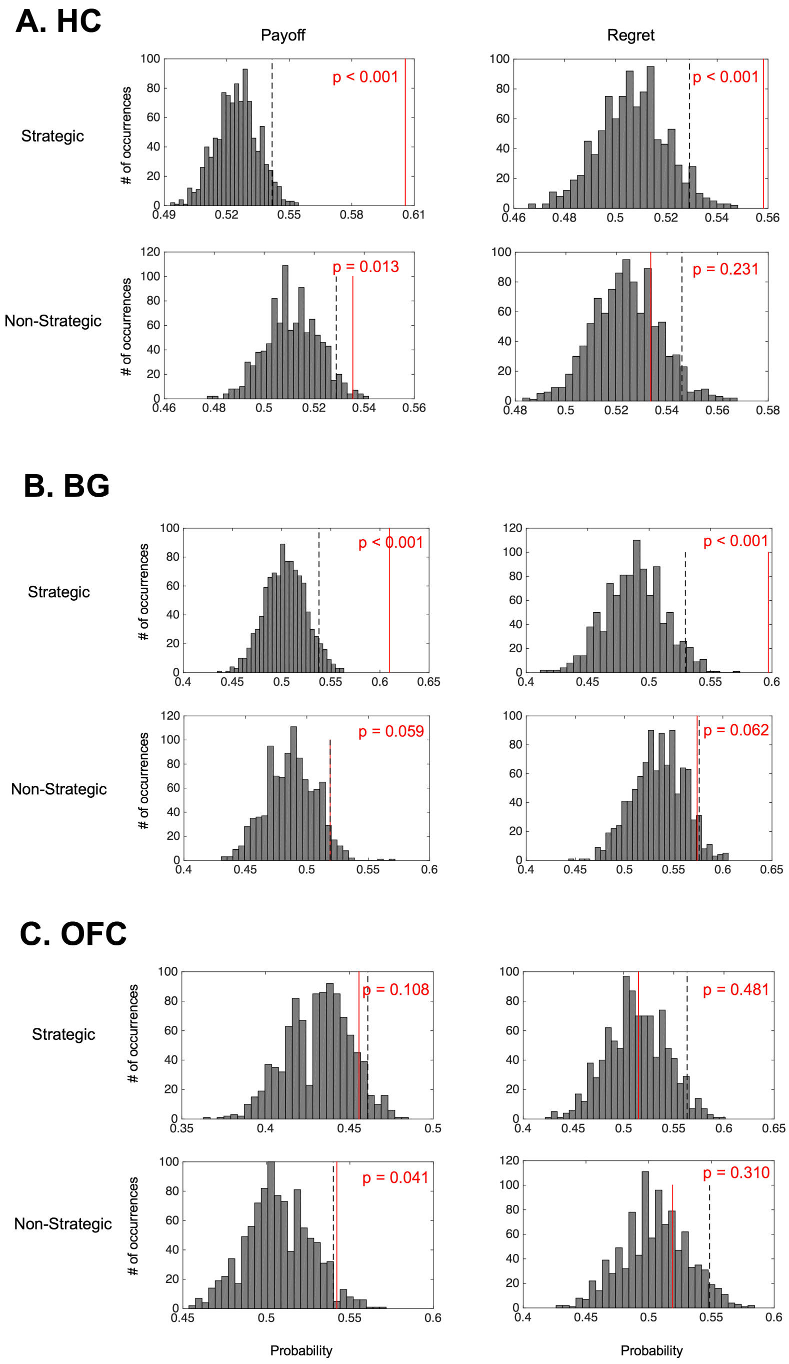

Supplementary Figure 2. Permutation test for the stay/switch pattern conditional on the level of payoff (left) or regret (right) for the cohort of HC (**A**), BG (**B**), and OFC (**C**), respectively. Histograms depict numbers of occurrences of different proportion of trials where participants stayed with the same decision if the payoff was high or the regret was low (according to a median split within each cohort and each condition) and switched to alternative actions otherwise, based on 1,000 randomly shuffled choice sequences within each subject. Dashed lines indicate 95% bounds of permutated distributions. Red lines represent the observed value.

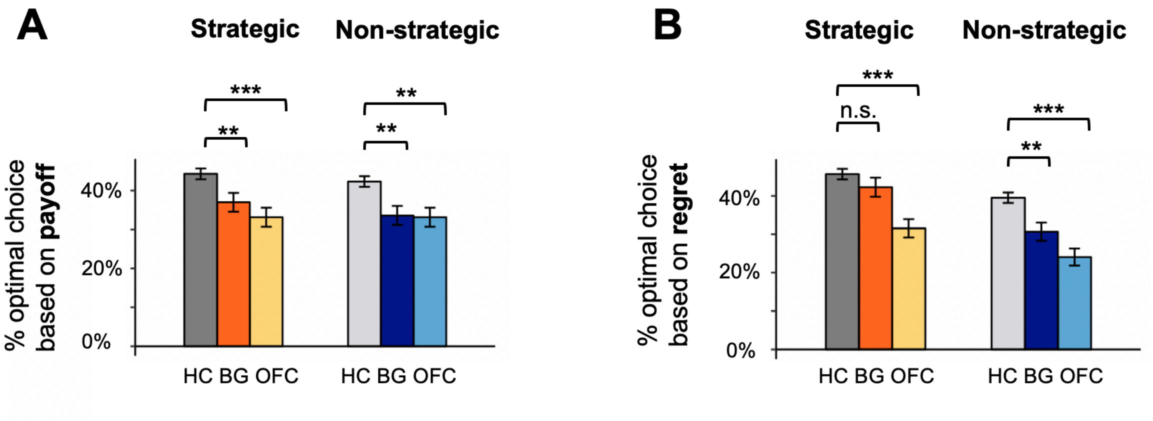

Supplementary Figure 3. The percentage of choosing optimal actions based on two model-free measures of optimal choices constructed using the history of payoff **(A)** or regret **(B)**. By comparing the average payoff (or regret) associated with each action within the past 10 trials, we calculated the proportion of trials in which actions with the highest average payoff (or lowest average regret) were selected. Complementary to the frequency of switching or staying given payoff/regret in **Fig. 2b**, these model-free measures demonstrate whether participants were switching to more or less adaptive strategies in the course of learning. Z-test for two proportions, * $P < .05$, ** $P < .01$, *** $P < .001$. Error bars represent S.E.M.

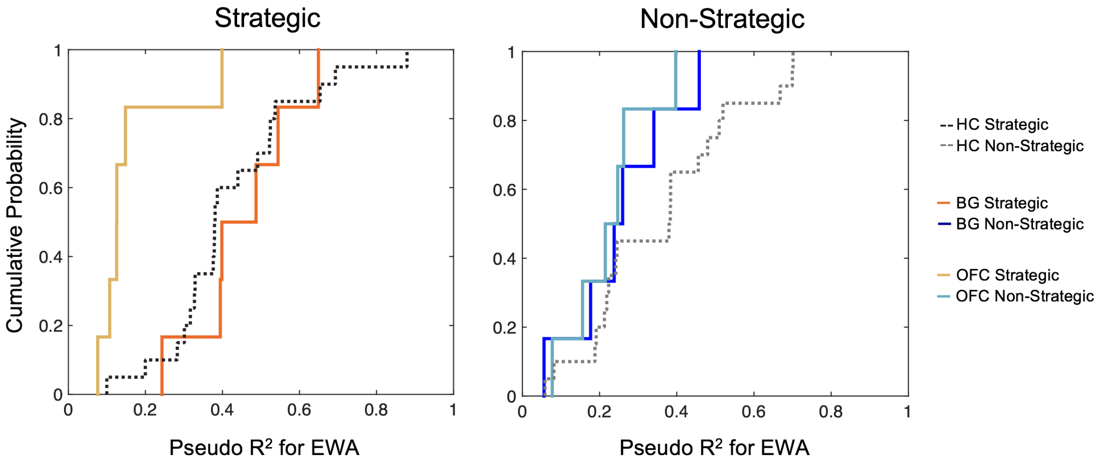

Supplementary Figure 4. Distribution of pseudo-R^2 at the individual level. To illustrate individual differences in how much EWA outperformed random choice model in fitting each subject's data, we calculated the empirical cumulative distributions (ECDF) for pseudo-R^2 within each cohort and each condition. This highlights distributional differences at all quantiles, and shows that the goodness of EWA model fit of BG cohort was indissociable with that of healthy controls in strategic condition, but not the non-strategic condition.

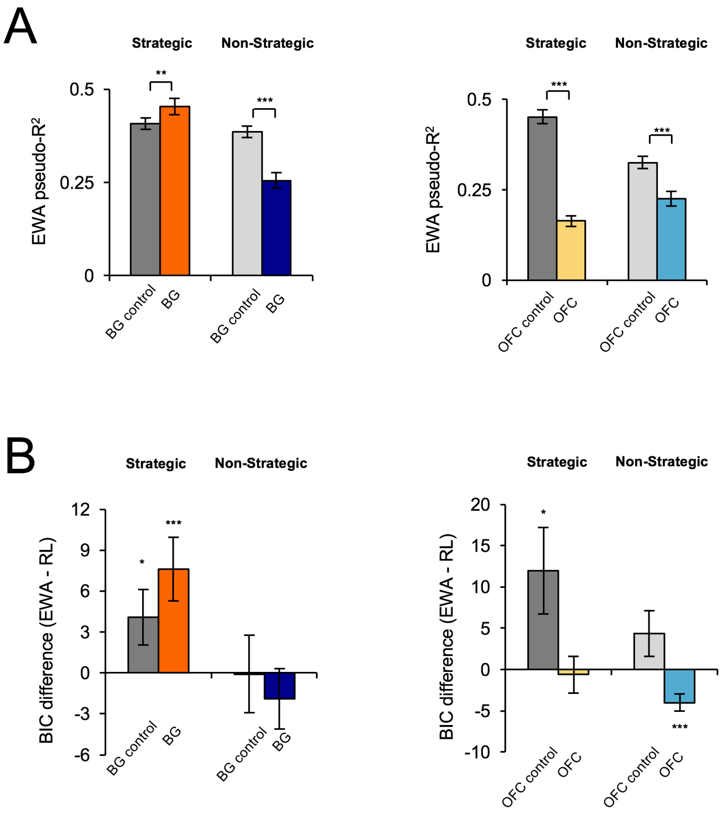

Supplementary Figure 5. Choice behavior of patient cohorts in comparison with age-matched controls. **(A)** Identical to **Fig. 3a**, except HC cohorts have been partitioned such that they age-match patient cohorts on both mean and standard deviation. Consistent with results from the combined HC cohort, there was a significant cohort (BG vs. age-matched HC) by condition (strategic vs. non-strategic) interaction (Wilcoxon rank sum test, $P < 0.001$), such that, relative to BG controls, EWA provided worse fit for BG in the non-strategic condition (pseudo-R^2, BG controls: 0.39 ± 0.02, BG: 0.25 ± 0.02, Wilcoxon rank sum test, $P < 0.001$) yet slightly better fit in the strategic condition (pseudo-R^2, BG controls: 0.41 ± 0.01, BG: 0.45 ± 0.02, Wilcoxon rank sum test, $P = 0.008$). In contrast, the behavior of OFC patients was associated with significantly lower explainable variances than age-matched HC participants in both conditions (pseudo-R^2 in strategic condition, controls: 0.45 ± 0.02, OFC: 0.16 ± 0.02, Wilcoxon rank sum test, $P < 0.001$; pseudo-R^2 in non-strategic condition, controls: 0.33 ± 0.02, OFC: 0.23 ± 0.02, Wilcoxon rank sum test, $P < 0.001$), and a significant cohort by condition interaction (Wilcoxon rank sum test, $P < 0.001$), which was also in line with results from combined HC cohort. **(B)** Identical to **Fig. 3c**, except HC cohorts have been partitioned such that they age-match patient cohorts on both mean and standard deviation. Consistent with results from the combined HC cohort, in both BG control and OFC control cohorts, EWA significantly improves the fit only in the strategic (difference in BIC, BG control: 4.08 ± 2.03, bootstrap $P = 0.037$, OFC control: 11.98 ± 5.22, bootstrap $P = 0.023$) but not the non-strategic condition (difference in BIC, BG control: -0.08 ± 2.86, bootstrap $P = 0.940$, OFC control: 4.35 ± 2.81, bootstrap $P = 0.100$). Error bars represent S.E.M. $^{*}P < .05$; $^{**}P < .01$; $^{***}P < .001$

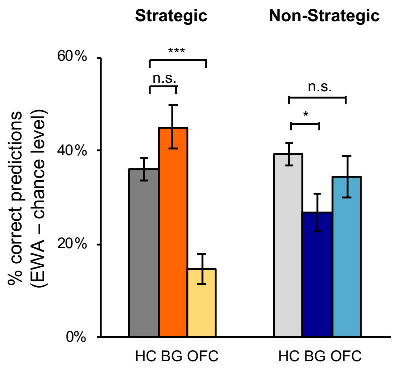

Supplementary Figure 6. Differential ability of EWA in explaining choice behavior as measured by the out-of-sample prediction accuracy. To account for the potential issue of overfitting, we estimated the EWA model using data from the first 60 trials of each subject and tested on the last 20 trials to obtain the percentage of the correct prediction relative to a random choice model. Results were largely consistent with that based on the in-sample pseudo-R^2 as shown in **Fig. 3a**. In the strategic condition, there was no significant difference in how well EWA predicted choice behavior in BG and vs. HC cohorts (BG: $45\% \pm 5\%$, HC: $36\% \pm 2\%$, Fisher's exact test, $P = 0.086$), whereas in the non-strategic condition, the BG cohort was associated with lower out-of-sample predictability than that of HC (BG: $27\% \pm 4\%$, HC: $39\% \pm 2\%$, Fisher's exact test, $P = 0.013$). In contrast, the behavior of OFC patients was associated with lower out-of-sample predictability than HC participants in the strategic condition (OFC: $15\% \pm 3\%$, HC: $36\% \pm 2\%$, Fisher's exact test, $P < 0.001$) and similar prediction accuracy in the non-strategic condition (OFC: $34\% \pm 4\%$, HC: $39\% \pm 2\%$, Fisher's exact test, $P = 0.336$). Error bars represent S.E.M.

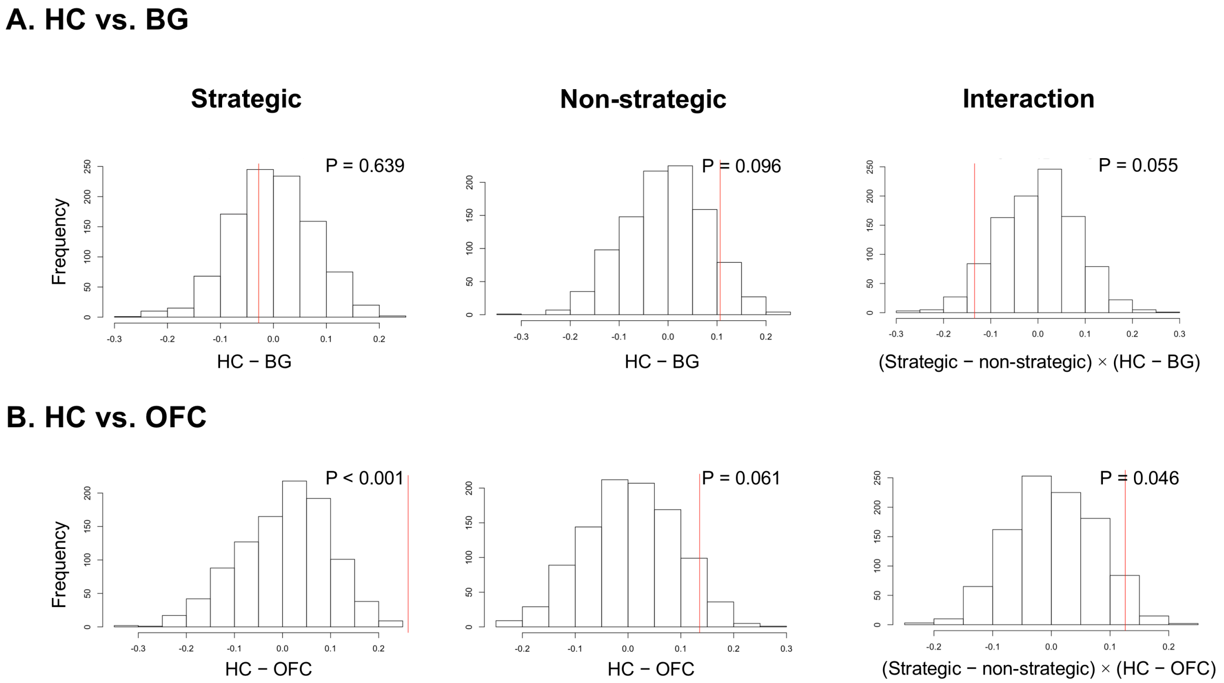

Supplementary Figure 7. Permutation tests on the difference of EWA pseudo-R^2 between HC and BG **(A)** or OFC **(B)** cohorts. To address the potential concern that the observed cohort differences in **Fig. 3a** was driven by individual differences in learning behavior rather than cohort differences, we randomly shuffled the group label between HC and each lesion cohort for 1,000 times, under each condition. The histograms depict null distributions of group mean differences for main effects of cohort under the strategic (left) and non-strategic (middle) conditions, as well as the interaction effect of cohort by condition (right). Largely consistent with results in **Fig. 3a**, there is no significant difference in terms of EWA model fit between BG and HC in the strategic condition, and a marginally significant interaction effect for (HC-BG) × (strategic – non-strategic).

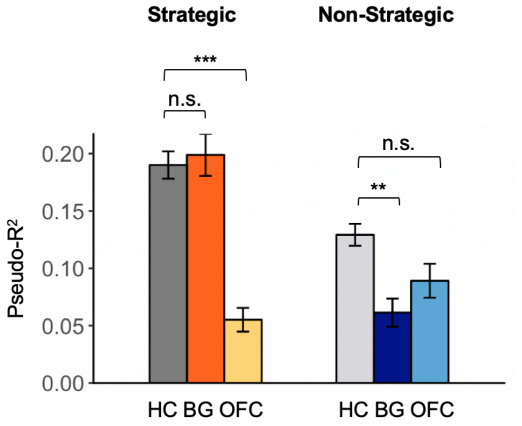

Supplementary Figure 8. Results of self-tuning EWA model fit. To evaluate the robustness of our results, we estimated self-tuning EWA model, which contains only two free parameters (i.e., inverse temperature and learning rate) reducing all other learning parameters into either fixed values or functions of experience (Ho et al., 2007). While the estimation results were largely consistent with that based on the standard EWA (as shown in Fig. 3A), the difference between OFC and HC cohorts in the non-strategic condition ceased to be insignificant, suggesting that OFC effects were sensitive to alternative model specifications. $^{*}P < .05$; $^{**}P < .01$; $^{***}P < .001$, Wilcoxon rank sum test. Error bars represent S.E.M.

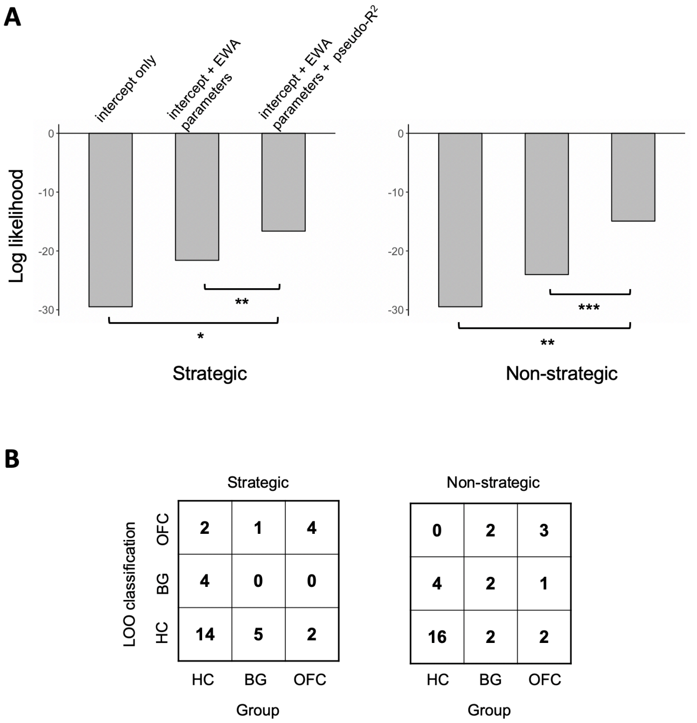

Supplementary Figure 9. Classification of subjects' lesion labels (BG vs. OFC vs. HC) based on inputs derived from EWA estimation. Three multinomial logistic regression models that predict individual labels were fit and compared. The first model, which serves as a benchmark, included only an intercept term to account for different sample sizes across cohorts. The second model, which serves to test the predictive power of EWA parameters, contained in addition the estimated parameters of each individual based on the best fitting EWA. The third model, which serves to test whether information related to model fits can predict brain lesion category above and beyond parameter estimates, contained in addition the value of pseudo-R^2 for each individual. **(A)** Model comparison based on likelihood ratio tests. **(B)** Classification performances of the winning multinomial model using a leave-one-out (LOO) procedure. Consistent with findings that BG patients performed similarly to HC in the strategic condition, the classifier did particularly poorly in distinguishing between BG and HC in the strategic environment, mislabeling 5 out of 6 BG patients as HC. On the other hand, in the non-strategic condition, the model made fewer mistakes in distinguishing between HC and the lesion patients, consistent with findings that HC outperformed both patient cohorts in the non-strategic environment. $^{*}P < .05$; $^{**}P < .01$; $^{***}P < .001$.

A

	Strategic		*Non-Strategic*	
	ΔAIC[1]	Likelihood ratio test	ΔAIC	Likelihood ratio test
HC	48.51	$\chi^2(6) = 60.51, p < 0.001$	11.75	$\chi^2(6) = 23.75, p < 0.001$
BG	34.87	$\chi^2(6) = 46.87, p < 0.001$	-0.63	$\chi^2(6) = 11.37, p = 0.078$
OFC	-5.78	$\chi^2(6) = 6.22, p = 0.399$	4.33	$\chi^2(6) = 16.33, p = 0.012$

[1]ΔAIC: AIC of reduced model – AIC of full model, positive value means the full model is better

B

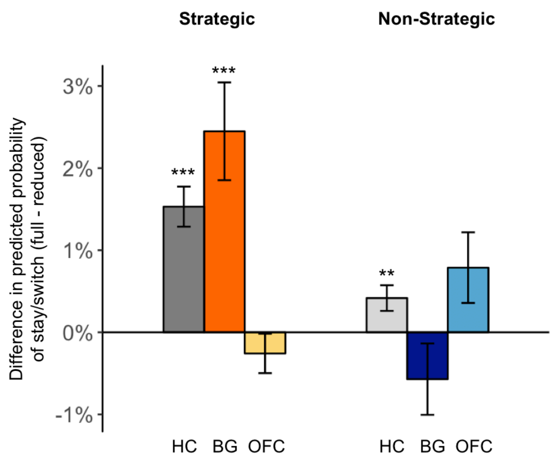

Supplementary Figure 10. Choice predictability as measured by logistic regression. As a robustness check, we examined the learning performance of participants using a model-free approach that does not depend on specific assumptions of the functional form such as EWA. Specifically, we examined the predictability of stay/switch decisions, differentiating between two sources of explanatory variables: those related to the characteristics of subjects and tasks, and those emerged endogenously from the dynamic interaction against opponents. If subjects demonstrate some type of learning, their decisions should be influenced to some extent by some endogenous signals arising from past interactions. That is, the set of endogenous variables should be able to predict decisions above and beyond the set of characteristic variables, if there is learning. We thus evaluated the predictive power of these two sets of variables by comparing a reduced and a full model based on either the in-sample goodness-of-fit measures **(A)** and the out-of-sample prediction accuracy using the leave-one-subject-out procedure **(B)**. The independent variables in the reduced model contained basic individual and task level features such as age, gender, the amount of endowment, and trial number. The full model additionally contained 6 variables including information of past 2 trials regarding the amount of payoff, regret, and the opponent's action. Standard fixed-effect logistic regression on stay/switch decisions was performed for each cohort in each condition separately, and demonstrated results largely consistent with model-based analyses shown in **Fig. 3a**. Error bars represent S.E.M. $^{*}P < .05$; $^{**}P < .01$; $^{***}P < .001$.

Supplementary Table 1. Demographic information of lesion cohorts and healthy controls. The HC subjects have been partitioned into BG controls and OFC controls, such that they age-match patient cohorts on both mean and standard deviation. Also see **Supplementary Figure 5** for the choice behavior of patient cohorts in comparison with age-matched controls.

Cohort	*N*	*Age*	*Gender (F)*	*Years of education*	*Estimated WAIS*[1]	*Bower Score*	*Etiology*	*Hemisphere*
BG lesion	6	63.83 (8.84)	2	13.33 (1.75)	103.83 (9.28)	4.33 (3.50)	Stroke (5) Hypertensive bleed (1)	Left (4) Right (2)
OFC lesion	6	42.67 (14.11)	3	14.67 (2.80)	106.33 (7.74)	9.83 (5.49)	Traumatic brain injury[2] (5) Tumor resection (1)	Bilateral (6)
Health comparison	20	56.60 (15.42)	12	16.55 (1.96)	114.30 (9.16)	10.79 (5.56)	NA	NA
BG comparison	12	67.25 (5.43)	9	16.08 (2.02)	116.75 (9.94)	9.09 (4.83)	NA	NA
OFC comparison	8	40.63 (10.62)	3	17.25 (1.75)	110.63 (6.84)	13.13 (5.65)	NA	NA

Parentheses contain standard deviations.

[1] WAIS: Wechsler Adult Intelligence Scale. WAIS scores were estimated from Shipley Institute of Living Scale.

[2] All TBI patients had low impact force injuries with no clinical or MRI evidence of axonal shear.

Supplementary Table 2. (Top) Companion to **Table 1**: Logistic regressions testing for the extent to which subjects' choice behavior was guided by payoffs and regrets. **(Bottom)** Companion to **Fig. 3a**: Comparison of differences in EWA pseudo-R^2 between HC and each lesion cohort, after controlling for age, gender, and education, as well as neuropsychological assessments such as scores of Wechsler Adult Intelligence Scale and Bower test.

	Strategic			***Non-strategic***		
	HC	BG	OFC	HC	BG	OFC
Payoff	1.17***	1.11***	0.11	0.54***	0.40	0.71**
	(0.12)	(0.26)	(0.23)	(0.11)	(0.21)	(0.21)
Regret	-0.48***	-0.99***	-0.02	-0.19	-0.30	-0.19
	(0.11)	(0.21)	(0.21)	(0.11)	(0.21)	(0.21)

	Strategic	***Non-Strategic***
HC vs. BG	0.01<br>(0.03)	0.18***<br>(0.03)
HC vs. OFC	0.34***<br>(0.03)	0.19***<br>(0.03)

Parentheses contain standard error of mean.

Bonferroni corrected, * $P < .05$; ** $P < .01$; *** $P < .001$

Supplementary Table 3. Summary statistics of fitted parameters based on EWA and RL models for lesion and healthy control cohorts.

	Strategic			*Non-Strategic*		
	HC	BG	OFC	HC	BG	OFC
**EWA**						
λ	3.16<br>(7.24)	0.47<br>(0.70)	1.3<br>(1.92)	1.17<br>(1.74)	0.9<br>(1.30)	0.11<br>(0.15)
ρ	0.69<br>(0.40)	0.35<br>(0.48)	0.79<br>(0.39)	0.79<br>(0.33)	0.7<br>(0.36)	0.64<br>(0.34)
δ	0.7<br>(0.38)	0.7<br>(0.46)	0.59<br>(0.47)	0.45<br>(0.47)	0.18<br>(0.40)	0.58<br>(0.49)
$N(0)$	75.3<br>(38.85)	95.35<br>(11.40)	53.6<br>(51.43)	79.47<br>(29.83)	92.5<br>(18.37)	83.47<br>(25.61)
ϕ	0.87<br>(0.24)	0.82<br>(0.40)	0.83<br>(0.26)	0.9<br>(0.21)	0.94<br>(0.05)	0.92<br>(0.20)
**RL**						
λ	0.10<br>(0.13)	0.18<br>(0.33)	0.05<br>(0.03)	0.07<br>(0.09)	0.03<br>(0.03)	0.04<br>(0.04)
ϕ	0.83<br>(0.27)	0.78<br>(0.39)	0.63<br>(0.45)	0.87<br>(0.19)	0.95<br>(0.03)	0.96<br>(0.05)

Parentheses contain standard deviations.

Supplementary Table 4. EWA pseudo-R^2 was not correlated with individual demographic variables or neuropsychological assessments across either conditions (upper) or cohorts (lower). Reported in the table are Spearman's ρ and p-values.

Across conditions, EWA pseudo-R^2 is not correlated with individual demographic variables

	Strategic		***Non-Strategic***	
	ρ	P	ρ	P
Age	0.11	0.540	0.06	0.762
Years of education	-0.07	0.711	0.09	0.607
IQ	0.02	0.918	0.08	0.664
Score of Bower test	0.04	0.811	0.21	0.252

Across cohorts, EWA pseudo-R^2 is not correlated with individual demographic variables

	HC		***BG***		***OFC***	
	ρ	P	ρ	P	ρ	P
Age	-0.07	0.681	-0.39	0.205	0.55	0.063
Years of education	-0.14	0.384	0.27	0.405	0.03	0.918
IQ	-0.17	0.296	0.16	0.629	0.44	0.154
Score of Bower test	0.24	0.146	0.44	0.154	-0.48	0.114

Supplementary Table 5. Across analyses, there was no significant association of EWA model fit and the lesion extent or location along the dorsal/ventral axis.

Null hypothesis	***Regression coefficient estimate***	
	Strategic condition	Non-strategic condition
There is no difference in EWA model fit between BG patients with and without lesion in the caudate	0.21 (0.393)[1]	-0.12 (0.740)
There is no difference in EWA model fit between BG patients with and without lesion in the globus pallidus	0.30 (0.323)	-0.04 (0.914)
There is no difference in EWA model fit between BG patients with and without lesion extending to the insular cortex	0.33 (0.226)	-0.09 (0.766)
There is no association of EWA model fit with lesion volume in BG	0.00 (0.751)	0.01 (0.615)
There is no difference in the degree of EWA improvement over RL in BG patients with and without lesion in the caudate	0.01 (0.919)	0.01 (0.687)
There is no difference in the degree of EWA improvement over RL in BG patients with and without lesion in the globus pallidus	0.02 (0.828)	0.00 (0.889)
There is no difference in the degree of EWA improvement over RL in BG patients with and without lesion extending to the insular cortex	0.01 (0.895)	-0.04 (0.086)
There is no association of the degree of EWA improvement over RL with lesion volume in BG	0.00 (0.833)	0.00 (0.725)

[1]Parentheses contain p-values.

Supplementary Table 6. Findings were robust to exclusion of patients with caudate lesions. All test statistics are Bonferroni corrected.

Null hypothesis	***Statistics***
EWA fit similarly in BG vs. HC under strategic condition as measured by pseudo-R^2	Wilcoxon rank sum test, W = 256920, p = 0.920
EWA fit similarly in BG vs. HC under non-strategic condition as measured by pseudo-R^2	Wilcoxon rank sum test, W = 291570, p < 0.01
There is no interaction between cohort (BG vs. HC) and condition (strategic vs. non-strategic) in terms of EWA model fit as measured by pseudo-R^2	Wilcoxon rank sum test, W = 222340, p < 0.01
EWA did not explain the choice behavior above and beyond RL for BG in strategic condition as measured by BIC difference	Bootstrap 95% CI: (3.73, 8.94)
EWA did not explain the choice behavior above and beyond RL for BG in non-strategic condition as measured by BIC difference	Bootstrap 95% CI: (-4.42, 2.80)
