# Supplementary material for: Patients with basal ganglia damage show preserved learning in an economic game
Source: Nat Commun. 2019 Feb 18;10:802. doi: 10.1038/s41467-019-08766-1 (PMC6379550; doi:10.1038/s41467-019-08766-1)
Supplement: Supplementary file 3 — Description of Additional Supplementary Files [file 41467_2019_8766_MOESM3_ESM.pdf]

Description of Supplementary Files

File Name: Supplementary Data 1
Description: Behavioral data from 24 healthy participants in the Patent Race Game. These were used as pilot data for power calculations referred to in the Methods section. They were also used as the "pool players" behavior referred to in the main text.
